# Wastewater surveillance in the COVID-19 post-emergency pandemic period: A promising approach to monitor and predict SARS-CoV-2 surges and evolution

**DOI:** 10.1016/j.heliyon.2023.e22356

**Published:** 2023-11-17

**Authors:** Bryan Sanchez Jimenez, Trinity Sterling, Austin Brown, Brian Modica, Kaylee Gibson, Hannah Collins, Carolyn Koch, Tyler Schwarz, Kristine N. Dye

**Affiliations:** a Department of Health Sciences, Stetson University, DeLand, FL, 32723, USA; b Department of Biology, Stetson University, DeLand, FL, 32723, USA

## Abstract

On May 24, 2023, approximately 3.5 years into the pandemic, the World Health Organization (WHO) declared the end of the COVID-19 global health emergency. However, as there are still ∼3000 COVID-19 deaths per day in May 2023, robust surveillance systems are still warranted to return to normalcy in times of low risk and respond appropriately in times of high risk. The different phases of the pandemic have been defined by infection numbers and variants, both of which have been determined through clinical tests that are subject to many biases. Unfortunately, the end of the COVID-19 emergency threatens to exasperate these biases, thereby warranting alternative tracking methods. We hypothesized that wastewater surveillance could be used as a more accurate and comprehensive method to track SARS-CoV-2 in the post-emergency pandemic period (PEPP). SARS-CoV-2 was quantified and sequenced from wastewater between June 2022 and March 2023 to research the anticipated 2022/23 winter surge. However, in the 2022/23 winter, there was lower-than-expected SARS-CoV-2 circulation, which was hypothesized to be due to diagnostic testing biases but was confirmed by our wastewater analysis, thereby emphasizing the unpredictable nature of SARS-CoV-2 surges while also questioning its winter seasonality. Even in times of low baseline circulation, we found wastewater surveillance to be sensitive enough to detect minor changes in circulation levels ∼30–46 days prior to diagnostic tests, suggesting that wastewater surveillance may be a more appropriate early warning system to prepare for unpredictable surges in the PEPP. Furthermore, sequencing of wastewater detected variants of concern that were positively correlated with clinical samples and also provided a method to identify mutations with a high likelihood of appearing in future variants, necessary for updating vaccines and therapeutics prior to novel variant circulation. Together, these data highlight the effectiveness of wastewater surveillance in the PEPP to limit the global health burden of SARS-CoV-2 due to increases in circulation and/or viral evolution.

## Graphical abstract

**Figure:**
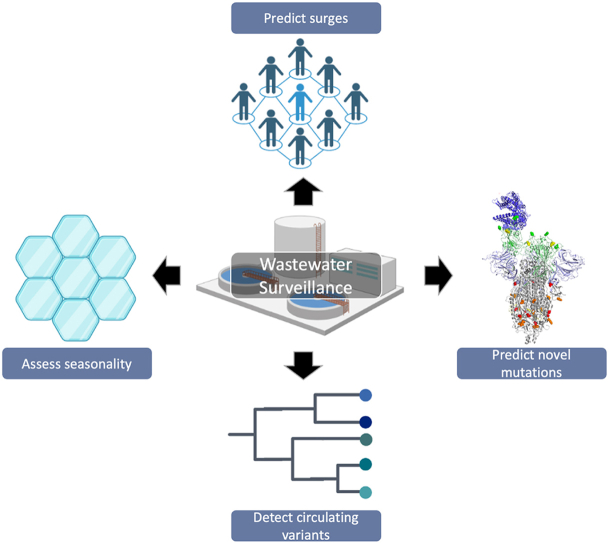

## 1 Introduction

Approximately three years after the start of the COVID-19 pandemic, the World Health Organization (WHO) officially declared the end of the COVID-19 global health emergency on May 4, 2023 [1]. As of May 2023, the SARS-CoV-2 pandemic is estimated to be directly responsible for over 766 million infections, resulting in greater than 6.9 million deaths [2]. Although no longer a global health emergency, as of May 2023 COVID-19 still amounts to over 400,000 new cases and greater than 3000 deaths per week worldwide [2]. These deaths, despite widespread immunity, are likely a result of a constant influx of aging individuals into high-risk populations and the development of comorbidities [3,4]. Therefore, although this announcement indicates that the population has entered the post-emergency pandemic period (PEPP), it does not signify the end of the COVID-19 burden on global health. In the PEPP, society must have the capability to ascertain periods of low risk in which individuals can continue normal behaviors while also predict periods of high risk in which society can adopt appropriate mitigation strategies.

Pandemic tracking statistics, including those reported above, rely heavily on clinical diagnostic tests. However, the role of clinical diagnostic tests in SARS-CoV-2 monitoring has been limited by several biases that have evolved over the course of the pandemic, such as their development and distribution, governmental guidelines, personal beliefs, low reporting of positive at-home rapid antigen tests, an increase in false negatives, undiagnosed asymptomatic cases, and pandemic fatigue [[5], [6], [7], [8], [9]]. Therefore, it is likely that the statistics generated by diagnostic tests underrepresent the true number of SARS-CoV-2 infections and circulation. Furthermore, as society has reached the end of the COVID-19 global health emergency, it is likely that the biases that have already impaired society's ability to perform tracking and prediction of SARS-CoV-2 infection levels and evolution will be exasperated. Therefore, the WHO, national, and supranational organizations, such as the United States Center for Disease Control and Prevention (CDC) and the European Commission, respectively, have recently encouraged the use of alternative methods of pandemic tracking, such as wastewater surveillance in the PEPP [10,11].

Although wastewater testing has been used to surveil other pathogens, such as poliovirus, for decades, it was not initially considered an option for SARS-CoV-2 as it is primarily a respiratory disease [12,13]. However, it was found that despite being a respiratory pathogen, SARS-CoV-2 is shed into wastewater [[14], [15], [16]]. Further, individuals infected with SARS-CoV-2 shed the virus into wastewater regardless of their personal biases, access to diagnostic tests, and vaccination status. Therefore, although diagnostic SARS-CoV-2 tests are necessary for individual health decisions, wastewater surveillance is a more accurate, comprehensive, and predictive measure of community circulation necessary for pandemic tracking, especially in the PEPP [15,17,[18], [19], [20]].

Beyond personal health decisions, clinical diagnostic tests have also been a source of patient samples to be used for sequencing to study viral evolution and detect variants of concern (VOC) [21]. SARS-CoV-2 contains a ∼30 kb genome that encodes 25 non-structural and 4 structural proteins, including the spike (S), nucleocapsid (N), membrane (M), and envelope (E) proteins [22]. The S protein of SARS-CoV-2 is the most extensively studied as it is responsible for binding to the host cellular ACE-2 receptor through its receptor binding domain (RBD), which allows for viral entry and infection of host cells necessary to propagate the infection [23]. Random mutations affecting this protein will be either positively or negatively selected based on how they affect the ability of the virus to replicate and transmit [24]. VOCs exhibit mutations that allow the virus to escape the host immune system, particularly neutralizing antibody responses that aim to prevent viral entry by blocking the RBD ACE-2 interaction [25]. As immunity against the current VOC increases in the population, natural selection will positively select for mutations that can evade these immune responses giving rise to novel VOCs [26].

The tracking of SARS-CoV-2 VOCs and their mutations is primarily accomplished by sequencing a subset of clinical diagnostic positive samples or allele-specific wastewater qRT-PCR [21,27]. Mutations that increase in frequency over time are likely advantageous and increase viral fitness, whereas mutations that maintain low frequency or disappear over time are likely neutral or deleterious. As robust clinical testing is necessary to correctly assess mutation frequency, the end of the COVID-19 global health emergency, and the consequent decrease in clinical testing, threatens to further limit these analyses, thereby promoting the use of alternative sequencing methods such as wastewater surveillance. Furthermore, as many therapies are reliant on SARS-CoV-2 sequences, including vaccines and monoclonal antibodies, tracking SARS-CoV-2 evolution through wastewater may provide insight into the effectiveness of these therapies prior to new VOC appearance [28,29].

Environmental surveillance of SARS-CoV-2 in wastewater may prove an effective and less biased model of pandemic tracking as the intensity of clinical testing is scaled down at the end of the global health emergency, consistent with the recommendation from the WHO [30]. However, as most of the studies aimed to gauge the effectiveness of SARS-CoV-2 wastewater surveillance have occurred during pandemic surges and in combination with a robust clinical diagnostic testing data set, it is unknown how well SARS-CoV-2 wastewater surveillance can perform on its own with less testing, and possibly in periods of low SARS-CoV-2 circulation in the PEPP. Therefore, the specific objectives of this study were to (1) assess the effectiveness of environmental wastewater SARS-CoV-2 surveillance and its ability to overcome biases associated with clinical diagnostic testing, (2) confirm or deny unexpected levels of infection at time points late in the pandemic, (3) assess its ability to predict future changes in infection levels in times of low baseline circulation in the PEPP, (4) independently detect predominant variants of concern, and (5) predict novel mutations that may be found in future variants of concern.

## 2 Materials and methods

### 2.1 Study location and wastewater sample collection

**Fig. 1:**
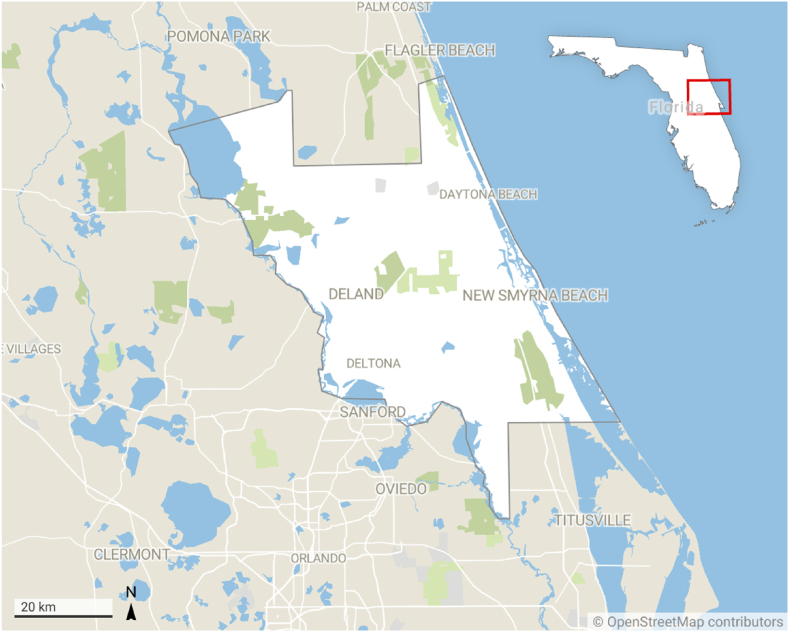
Wastewater samples collected from a DeLand WWTP that services Volusia County, Florida. Created with Datawrapper.

From June 2022 to March 2023, 200 mL, 24-h, composite, influent wastewater samples were collected every two weeks from a WWTP that contains 14,000 service connections and services between 33,000 and 35,000 residents throughout the majority of Volusia County, Florida (Fig. 1 and Supplementary Fig. 1). Sample collection from the facility autosampler was completed at ∼7:00 a.m. for every time point, and was transported at 4 °C to the laboratory for immediate processing.

### 2.2 Wastewater processing, pasteurization, precipitation, and RNA extraction

Wastewater processing, pasteurization, precipitation, and RNA extraction were performed as described previously, with several modifications [[31], [32], [33]]. Wastewater samples were pasteurized in their original container at 60 °C for 60 min, starting when the sample reached 60 °C. Solids separation was performed by a 5000×*g*, 10-min centrifugation, and the resultant supernatant was filtered through a 0.22 μM filter. A PEG/NaCl viral precipitation was then performed for 48 h at 4 °C. After the viral precipitation was complete, the sample was stained with a 2 % safranin solution prior to a 2-h, 12,000×*g* centrifugation to pellet the virus before decanting the supernatant. The safranin dye stained the viral pellet making it more easily visualized and able to be avoided when removing the supernatant. RNA extraction was performed by first resuspending the pellet in TRIzol, followed by phase separation with chloroform. The resultant sample was then centrifuged at 12,000×*g* for 15 min at 4 °C. After centrifugation, the aqueous phase was removed, and isopropanol was used to precipitate the RNA, followed by ethanol washes, air-drying of the RNA pellet, and elution in nuclease-free water. Samples were then aliquoted into tubes for SARS-CoV-2 RT-qPCR, PMMoV RT-qPCR, or whole genome sequencing before being stored at −80 °C.

### 2.3 RT-qPCR detection of SARS-CoV-2 N1 and PMMoV

Detection of SARS-CoV-2 N1 and PMMoV was performed as described previously with some modifications [[31], [32], [33]]; F [22]. To detect and quantify SARS-CoV-2 N1 and PMMoV RNA in our sample, we performed RT-qPCR using the Applied Biosystems StepOne RT-qPCR System (ThermoFisher Scientific). Wastewater RNA, positive controls, and negative controls were run in duplicate with a master mix containing 2× OneStep RT-PCR Buffer, TaKaRa Ex Taq HS, PrimeScript RT Enzyme Mix, High Rox Dye, and RNAse Free Water which are all supplied in the One Step PrimeScript RT-PCR Kit (TaKaRa). Additionally, the master mix contained either SARS-CoV-2 N1 Primers/Probe Kit (IDT) for SARS-CoV-2 detection or custom-made primers and probes for PMMoV detection as described previously [32]. A standard curve for SARS-CoV-2 was created by performing a 1:10 serial dilution of the already 1:100 diluted EURM019 positive control (Millipore Sigma). A standard curve for PMMoV was performed using a serial dilution of a PMMoV oligo as described previously [32]. Non-template, negative controls contained nuclease-free water instead of a positive control or wastewater sample. All samples for SARS-CoV-2 or PMMoV were loaded into a 48-well Reaction Plate (MidSci) and were run at the following cycling conditions: SARS-CoV-2 N1: 25 °C for 2 min, 50 °C for 15 min, 95 °C for 2 min, followed by 45-cycles of 95 °C for 3 s and 55 °C for 30 s. PMMoV: 25 °C for 2 min, 50 °C for 15 min, 95 °C for 2 min, 95 °C for 10 min, followed by 45-cycles of 95 °C for 15 s and 57 °C for 1 min.

### 2.4 Extrapolation of sample concentration from the standard curve

Following RT-qPCR for both SARS-CoV-2 N1 and PMMoV, cycle thresholds (Ct) for each standard, unknown, and non-template control were confirmed to be within 0.2 standard deviations of their duplicates. Time point data was only used if non-template controls were negative for both SARS-CoV-2 N1 and PMMoV. Known concentrations of positive control standards were plotted against their Ct, followed by extrapolating the unknown wastewater sample concentration from its Ct.

### 2.5 Comparison of sample concentrations between different time points

As samples were collected at different time points over the course of several months, and it was important to receive data for our study in close to real-time, we were unable to run each sample on the same plate against the same standard, which is the gold standard when comparing the concentration between samples of different time points. Therefore, in order to compare the concentrations of each time point sample, which was extrapolated from separately prepared standard curves, the data from each time point was analyzed to be consistent with previous time points before acceptance. This included but was not limited to, ensuring amplification curves, final thresholds, Ct, and slope of each standard (SARS-CoV-2 and PMMoV) were within 10 % of other time point data. Any time point data that did not meet all of these criteria was omitted from the final analysis.

### 2.6 PMMoV normalization of SARS-CoV-2 concentration

SARS-CoV-2 concentrations were normalized using the PMMoV RT-qPCR data obtained from the same sample. In order to normalize, the following equation was used, as described previously[19].

$$Deviation\;Factor=\hat{10}\left[slope\;x\left(sample\;CT-median\;CT\right)\right]$$

$$\frac{SARS-CoV-2\;Sample\;Concentration}{Deviation\;Factor}$$

### 2.7 Whole genome sequencing

Viral RNAs extracted from wastewater samples were submitted to Azenta Life Sciences where they were reverse transcribed and amplified with SARS-CoV-2 primer sets tiling the whole viral genome. The resulting amplicons were sequenced using Illumina 2 × 150bp short read sequencing (∼1 M reads/sample) followed by bioinformatic data analysis.

### 2.8 Bioinformatic data analysis of whole genome sequencing

Raw reads (FASTQ files) were adapter and quality trimmed. Trimmed reads were then aligned to human reference genome hg38 first to remove potential host contamination. Reads after host subtraction were then mapped to SARS-CoV-2 reference genome (NC_045512.2) (https://www.ncbi.nlm.nih.gov/nuccore/1798174254). Primer sequences were soft-clipped based on alignment and the resulting bam files were used for genome consensus sequence generation and variant calling. For genome consensus calling, a minimum depth of 10 was required for a region to be considered and a minimum allele frequency of 0.5 was needed for a mutation to be called as the consensus bases for the genome position. SARS-CoV-2 lineages were classified using Pangolin (https://cov-lineages.org/). UShER was used for lineage inference, and then the strain of variants of concern were assigned by Scorpio. Clade and lineage classification were also inferenced using Nextclade (https://nextstrain.org/). The classification models are updated periodically.

## 3 Results and discussion

### 3.1 Wastewater surveillance of SARS-CoV-2 confirmed low infection levels in the winter of 2022/23

As of May 2023, over 550,000 people reside in Volusia County, Florida (Fig. 1), 63 % of whom are vaccinated against SARS-CoV-2 and 12 % of whom have received a bivalent booster [34]. Known for its tourism, Volusia County also welcomed 9.9 million tourists in 2021 alone [35]. Similar to other regions, Florida experienced a surge of COVID-19 cases in the winters of 2020, 2020/21, and 2021/22. These surges were largely expected, as it was hypothesized that SARS-CoV-2 would present as a winter seasonal pathogen similar to other respiratory viruses, including the common-cold coronaviruses [36,37]. Therefore, it was anticipated that another COVID-19 surge would occur during the winter of 2022/23. Furthermore, being the fourth winter of the COVID-19 pandemic, it was projected that pandemic fatigue and the widespread use of at-home rapid antigen tests may influence the number of tests performed and reported during the winter of 2022/23. This could obscure tracking efforts, in addition to other biases, and inhibit society's ability to determine if SARS-CoV-2 is continuing as a seasonal winter pathogen. Consequently, the absence of accurate SARS-CoV-2 tracking may give individuals a false sense of security, leading to more infections and worse disease outcomes [[38], [39]]. Therefore, we sought to track SARS-CoV-2 wastewater levels from June 2022 to March 2023, as we hypothesized that SARS-CoV-2 wastewater quantification would be a less biased representation of community infection levels necessary to observe SARS-CoV-2 continuing as a largely seasonal winter pathogen, and also inform individuals to adopt appropriate mitigation and safety precautions.

**Fig. 2:**
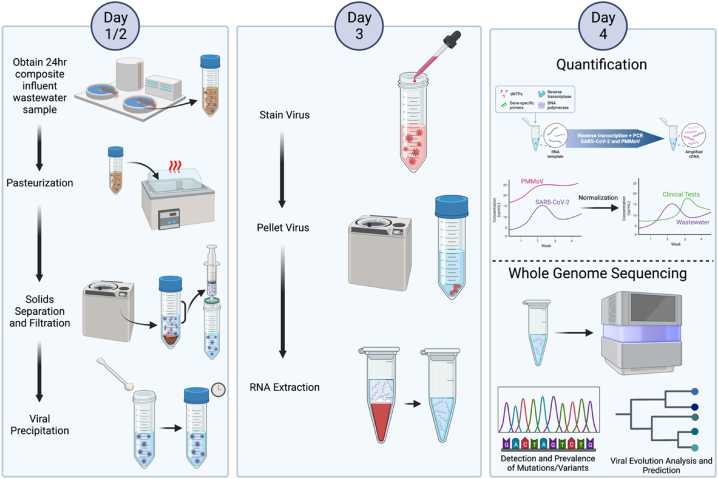
Graphical methods of wastewater sample collection and processing for SARS-CoV-2 N1 RT-qPCR and whole genome sequencing. Created with BioRender.com.

**Fig. 3:**
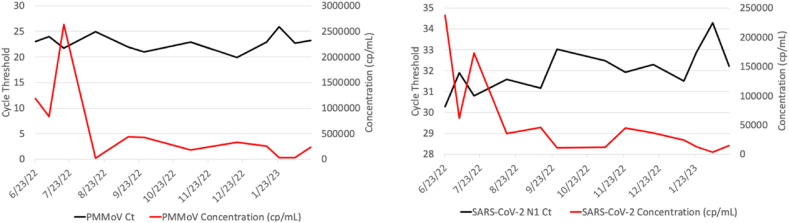
Cycle threshold and extrapolated concentration of SARS-CoV-2 and PMMoV in wastewater. The Ct (black) and extrapolated concentration (red) for every accepted wastewater sample for PMMoV (A) and SARS-CoV-2 N1 (B).

Wastewater samples obtained from a Volusia County, FL wastewater treatment plant (WWTP) were collected and processed in close to real-time for SARS-CoV-2 N1 RT-qPCR detection every two weeks between June 2022 and March 2023 (Fig. 2). As it is likely that the dilution rate of domestic wastewater can vary strongly between timepoints due to sewer inflow or runoff, industrial discharges, and extraneous water, we normalized the SARS-CoV-2 wastewater concentrations between time points with a consistently human shed plant pathogen, Pepper Mild Mottle Virus (PMMoV), which has been described previously [[20], [32]]. Indeed, the RT-qPCR Ct and extrapolated concentrations of PMMoV were found to be largely consistent over time, with only minor differences between time points (Fig. 3A), whereas the RT-qPCR Ct and extrapolated concentration of SARS-CoV-2 were found to have more profound differences between time points (Fig. 3B).

**Fig. 4:**
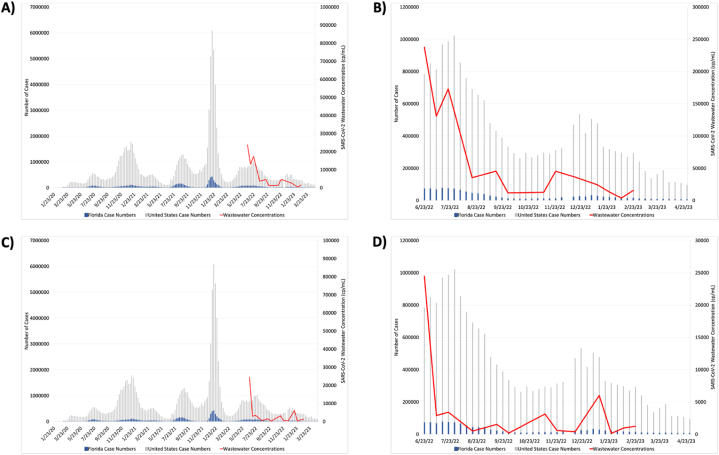
SARS-CoV-2 wastewater concentrations compared to clinical diagnostic tests. Clinical cases determined by test positivity in the United States (grey) and Florida (blue) compared to Volusia County, FL SARS-CoV-2 wastewater concentrations (red) both before (A and B) and after (C and D) normalization with PMMoV. A and C are showing the entire pandemic time period, whereas B and D are a showing the specific time period of our study.

At the conclusion of the 2022/23 winter, clinical testing unexpectedly did not observe another winter surge as anticipated from previous pandemic winter months and the knowledge of other respiratory pathogens. To ensure that this unexpected drop in the number of SARS-CoV-2 infections during the winter months was not a consequence of diagnostic testing biases, we compared our SARS-CoV-2 wastewater concentrations to clinical diagnostic tests in the months directly before, during, and after the winter of 2022/23. Surprisingly, our wastewater SARS-CoV-2 concentrations were positively correlated with clinical diagnostic tests in both the United States and Florida, showing a significant decline in SARS-CoV-2 wastewater concentrations beginning in August 2022 and continuing through March 2023, contrary to expectations of a winter surge (Fig. 4 A and B). Of note, there was a slight, transient increase in the number of positive clinical cases and wastewater concentrations in January 2023, which was detected by both diagnostic tests and wastewater surveillance.

To ensure that the decline in SARS-CoV-2 wastewater concentrations during the winter 2023 months was not a result of failing to normalize the data, we then normalized the SARS-CoV-2 concentrations against our PMMoV concentrations from each of the same time points and plotted the normalized SARS-CoV-2 concentrations against the United States and Florida case numbers determined by clinical diagnostic tests (Fig. 4C and D). Consistent with our previous findings, the normalized data also showed a rapid decline in SARS-CoV-2 wastewater concentrations during the same winter time period, further confirming the positive correlation between clinical diagnostic tests and wastewater surveillance, and also the unexpected decrease of infection during the winter of 2023.

Although as a society we are in the fourth year of SARS-CoV-2/COVID-19, its pandemic status, initially low population immunity, and limited initial research have impaired society's ability to make accurate predictions. For example, although the winters of 2020, 2020/21, and 2021/22 had SARS-CoV-2 surges similar to other respiratory pathogens, including the common-cold coronaviruses, this pattern did not continue as expected in the winter of 2022/23. Initially, it was hypothesized that this might be due to an increase in biases in the fourth winter of the pandemic; however, these results were verified by our environmental wastewater SARS-CoV-2 quantification. This close to real-time confirmation of low levels of circulation in times when a surge was expected is important as it allows individuals to confidently assume normal daily activities when the risk is low, necessary to return to normalcy, and increases the likelihood of adherence to mitigation strategies when appropriate. These data also prove society's limited ability to make accurate predictions, challenging the belief that SARS-CoV-2 is a consistent winter seasonal pathogen, and highlights the necessity of even more robust wastewater surveillance as society transitions into the PEPP.

### 3.2 SARS-CoV-2 wastewater quantification sensitivity permits prediction of changes in SARS-CoV-2 infection, even in times of low baseline circulation

The WHO has encouraged increased use of wastewater surveillance for SARS-CoV-2, not only due to its positive correlation with clinical diagnostic testing but also due to its ability to predict future surges, as has been reported previously [[40], [41], [42], [43]]. This predictive nature of wastewater quantification is important as it allows the public to confidently and safely assume normal behavior when levels are low and increase precautions only when necessary in order to mitigate spread and disease outcomes. However, as the winter of 2022/23 was a unique time with lower-than-expected infection levels, we sought to determine whether wastewater surveillance is sensitive enough to predict changes in community infection levels even when circulation is low, which would be imperative for wastewater SARS-CoV-2 quantification to be an effective early warning system at all times, especially in the PEPP. During the winter of 2022/23, there was a singular instance from December 9, 2022, through January 22, 2023, where diagnostic tests detected a minor increase in the number of infections. Similarly, we found that RT-qPCR quantification of SARS-CoV-2 RNA in wastewater was sensitive enough to predict this transient increase and decrease by approximately 30 and 46 days in the unnormalized and normalized wastewater data set, respectively (Fig. 4 A - D). Of note, the normalized data set also peaked 21 days after the peak determined by clinical tests. Although a positive and predictive correlation between wastewater surveillance and clinical testing was identified, the small data set and duration of gaps between time points made it difficult to differentiate between experimental noise and a legitimate increase, and determine an exact predictive interval. Therefore, to accurately rely on wastewater surveillance to predict future SARS-CoV-2 surges, it is necessary that the frequency of testing be increased.

As was seen in the summers of 2020, 2021, and 2022, it is possible that surges may occur at unexpected times. These previous surges were identified largely through increased test positivity, hospital admissions, and deaths. However, our results indicate that wastewater surveillance can predict even minor increases in real-time, importantly at low levels of baseline infection, approximately 30–46 days prior to clinical diagnostic tests. Therefore, at the conclusion of the COVID-19 global health emergency and consequent decline in clinical testing, wastewater surveillance can be used to predict unexpected surges necessary to implement timely mitigation strategies and public health resources.

### 3.3 SARS-CoV-2 wastewater surveillance is an appropriate method to detect variants of concern

**Fig. 5:**
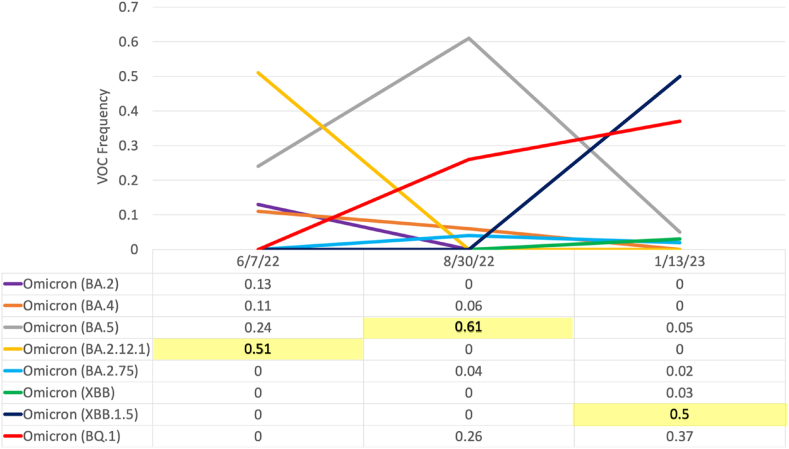
Comparison of VOC predominance in clinical versus wastewater samples. The frequency (0.5 equates to 50 %) of each variant of concern detected in clinical samples is shown both graphically and tabularly, with the predominant VOC detected in wastewater at each time point highlighted in yellow.

In addition to changes in the number of infections, different periods of the SARS-CoV-2 pandemic have been defined by viral evolution and the formation of variants. Currently, the majority of samples used for sequencing are obtained from diagnostic clinical tests [21]. However, as test usage is decreasing in the PEPP while also being subject to various biases, sequencing efforts to track viral evolution and detect VOCs must include alternative approaches such as the sequencing and allele-specific qRT-PCR of wastewater [27,44]. As VOCs have been positively selected for due to their increased ability to replicate and transmit compared to previous variants, they pose a significant health risk and therefore require continual tracking and research. Therefore, it is necessary to develop a method to overcome these biases and continue sequencing efforts even after the end of the global health emergency. For these reasons, we sought to determine whether whole genome sequencing (WGS) of SARS-CoV-2 RNA isolated from wastewater could detect similar VOCs to clinical respiratory samples. In order to test this, we performed WGS on a subset of wastewater RNA extractions at different time points (6/7/22, 8/30/22, 1/13/23) to determine the predominant VOC detected in wastewater compared to clinical samples. Indeed, we found that at each time point, the predominant VOC in clinical respiratory samples was the same as the predominant VOC sequenced from wastewater samples (Fig. 5). Of note, it is possible that future VOCs may have increased or reduced fecal shedding, as has been shown with reduced fecal shedding of the omicron variant, which may influence the correlation between wastewater variant prevalence and respiratory samples [45]. Together, these data found sequencing of SARS-CoV-2 RNA from wastewater to be an appropriate method to determine the predominant VOC in place of clinical samples as a result of reduced testing in the PEPP, and warrant future investigation with larger data sets.

### 3.4 Whole genome sequencing of SARS-CoV-2 isolated from wastewater can identify novel SARS-CoV-2 mutations and predict their likelihood of being found in future variants of concern

**Fig. 6:**
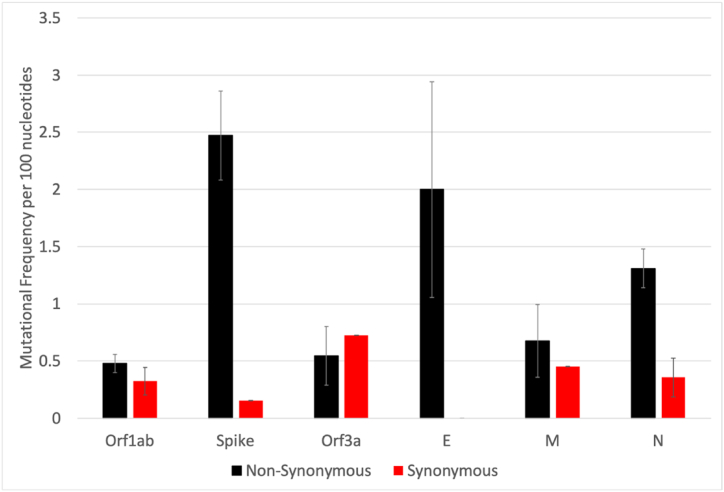
The number and frequency of synonymous and non-synonymous mutations per SARS-CoV-2 gene. WGS was performed on RNA extracted from wastewater, and the frequency of SARS-CoV-2 mutations in various genes and whether they are non-synonymous (black) or synonymous (red) is shown.

In addition to tracking known VOCs, it is also beneficial to identify novel mutations that may be found in future VOCs. The S protein of SARS-CoV-2 has been of particular interest for sequencing analyses as it is responsible for viral entry into the host cell and is, therefore, the predominant target of neutralizing antibody responses [23,24]. For these reasons, significant evolutionary pressure has been placed on the S protein to evade these antibody responses, in addition to other immunodominant structural proteins, including the E, M, and N proteins [46,47]. In order to determine whether sequencing of SARS-CoV-2 RNA isolated from wastewater could be used to observe SARS-CoV-2 evolution, we further analyzed our wastewater SARS-CoV-2 WGS results at two time points (6/21/22 and 1/13/23). Unexpectedly, our sequencing analyses allowed us to visualize the strong evolutionary pressure exerted on the SARS-CoV-2 immunodominant structural proteins, as the number of mutations per 100 nucleotides of the S, E, M, and N proteins were higher than that of the non-structural proteins, with the S gene having the greatest frequency of mutations (Fig. 6). Furthermore, instead of having an approximately equal number of synonymous or non-synonymous mutations, as was observed in other SARS-CoV-2 non-structural genes such Orf1ab, the mutations found in the S gene and other immunodominant non-structural genes were found to have a significantly higher number of non-synonymous mutations than synonymous. Although it is known that the SARS-CoV-2 RNA dependent RNA polymerase (RdRp) will randomly make synonymous and non-synonymous mutations across the genome uniformly, the unequal number and frequency of non-synonymous mutations in structural, immunodominant genes, such as the S gene, highlights the role of natural selection in evolution, as the necessity for non-structural, immunodominant proteins, such as S, to positively select for a large number of mutations that lead to protein changes to evade the immune system.

**Fig. 7:**
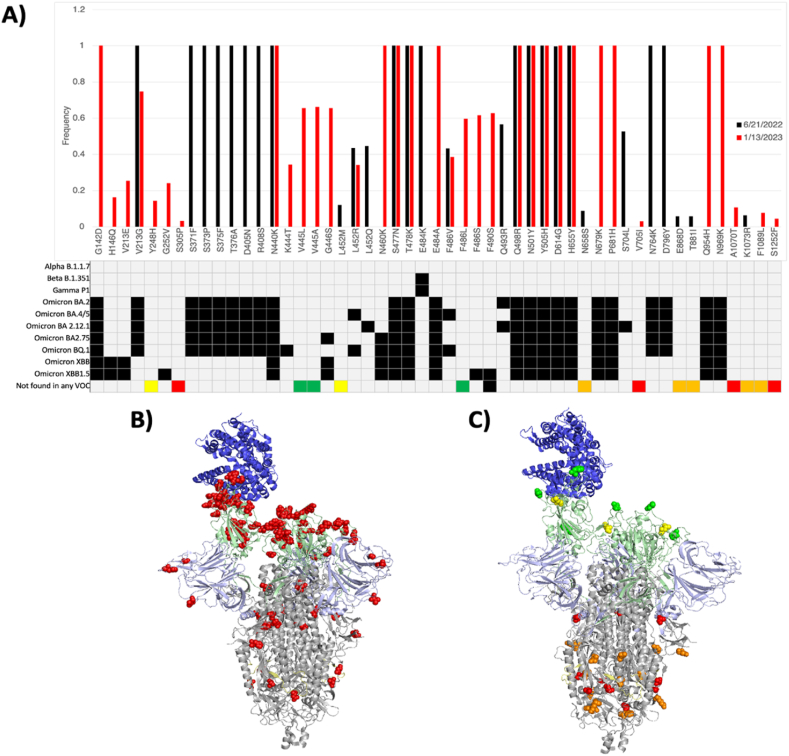
Whole genome sequencing of the SARS-CoV-2 S gene from wastewater. SARS-CoV-2 RNA extracted from wastewater was sequenced on 6/21/22 and 1/13/23. (A) Each mutation and its frequency on 6/21/22 (black) and 1/13/23 (red) were plotted. For each mutation, grey boxes designate variants of concern that do not contain the mutation and black boxes represent variants of concern that do contain the mutation. Colored boxes in the last row designate mutations that are not found in any variant of concern. The color of the box designates the frequency of the mutation, with red referring to 0–5%, orange 5–10 %, yellow 10–50 %, and green >50 %. The SARS-CoV-2 S protein contains multiple domains, including the receptor binding domain (light green), furin cleavage site (light yellow), and N-Terminal Domain (light blue) and the receptor binding domain (light green), which binds to the ACE-2 (dark blue) cellular receptor. Known (B) and novel (C) mutations were plotted as red spheres on the S protein structure, with novel mutations being color-coded based on their frequency, with red referring to 0–5%, orange 5–10 %, yellow 10–50 %, and green >50 %. SARS-CoV-2 S protein structures were created by PyMol.

We further analyzed our WGS data to evaluate non-synonymous mutations found in the S protein. As expected, we detected many mutations that have been characterized previously in specific VOCs (Fig. 7A). Furthermore, these mutations changed in frequency between time points, consistent with the predominant VOC at that time (Fig. 5). As expected, when these known mutations were plotted on the structure of the SARS-CoV-2 S protein, it was found that most amino acid changes clustered around the antigenic receptor binding domain (RBD), consistent with this region of the S protein exhibiting a strong selective pressure to evade neutralizing antibody responses in order to bind ACE-2 and infect a cell (Fig. 7B).

Interestingly, we also found 14 novel SARS-CoV-2 S mutations that have not been characterized previously (Fig. 7A). The frequency of these mutations varied across time points, suggesting implications for the selective benefit or disadvantage of each individual mutation and their consequent maintenance in future variants. Surprisingly, when these mutations were plotted on the structure of the SARS-CoV-2 S protein, the ones with the highest frequency, which are therefore the most likely to be advantageous and positively selected for, were found in the RBD, whereas the mutations with lower frequency, which are most likely to be deleterious or neutral and negatively selected for, were found towards the stalk of the S protein (Fig. 7C). Together, the frequency and position of amino acid changes may be used as a model to determine mutations with a high likelihood of being found in future VOCs, and should be further assessed as a potential method with larger, more complete, data sets. Furthermore, other SARS-CoV-2 proteins were also analyzed with similar results (Supplementary Table 1).

Monoclonal antibody therapy is a therapeutic in which an infected individual is infused with pre-made SARS-CoV-2 antibodies that usually bind various regions of the S protein RBD to prevent viral entry into host cells, with a significant efficacy against severe disease and death [48]. However, the use of this therapy has been impeded by the evolution of new variants [49]. For example, Sotrovimab was effective for Omicron BA.1, but had reduced efficacy against Omicron BA.2, BA.4, BA.5, and BA.2.12.1 [49]. The limited ability of clinical diagnostic testing to predict future mutations impaired the ability to research updated monoclonal antibody therapies effective for novel variants, thereby preventing individuals from receiving this treatment option for many months. Our results show that environmental wastewater surveillance is a more comprehensive, sensitive, and robust model that allows us to make data-driven predictions of future mutations when assessing the frequency and location of amino acid changes in the structure of the protein. Such analyses may prove influential in preparing for surges of novel variants and assessing the efficacy of vaccines and therapeutics. Also of note, because mutations and variants can arise in any area of the world, and environmental wastewater surveillance is easier implemented to surveil large and less-resourced areas, wastewater surveillance is more equipped for SARS-CoV-2 evolutionary studies than clinical diagnostic sampling, especially in light of the end of the COVID-19 global health emergency. Together, these data have found WGS of wastewater SARS-CoV-2 RNA to be a less-biased and more comprehensive method that is not only positively correlated with current variants of concern but may also provide a model to predict future mutations and variants that may be important for the updating of vaccines and therapeutics necessary for the preparation of future surges.

## 4 Conclusions

As the COVID-19 emergency concludes and society enters the PEPP, it is important to remain attentive to both SARS-CoV-2 infection levels and evolution. Even after widespread immunity elicited by natural infection and/or vaccination, as of May 24, 2023, ∼3000 individuals are dying per day of COVID-19, most likely as a result of the constant influx of individuals into high-risk populations and inequitable worldwide surveillance [50]. As surges are usually the result of contracting population immunity, human behavior, and/or new viral variants, safely returning to normal in the PEPP is reliant on a less biased and predictive surveillance system that can track each of these factors. However, the most utilized surveillance system to date relies on diagnostic testing, which is delayed in its surge warning and subject to many biases that are threatened to be exasperated in the PEPP. Conversely, wastewater surveillance has been found to be a reliable, less biased, and predictive tool to monitor SARS-CoV-2 infection levels in close to real-time. This is especially important in the PEPP, as SARS-CoV-2 has been found to be unpredictable in the timing of its surges, with wastewater surveillance confirming a lower-than-expected amount of circulation in central Florida in the 2022/23 winter. However, the ability of wastewater surveillance to detect minute changes 30–46 days prior to clinical diagnostic tests, even at low baseline levels of circulation, allows communities to confidently resume normal behaviors in periods of low risk, but preemptively adopt appropriate mitigation interventions when the risk increases, which will undoubtedly lead to less significant COVID-19 outcomes in the PEPP.

Wastewater surveillance also provides researchers with a less biased, comprehensive, and predictive tool to monitor viral evolution. In addition to identifying similar VOCs to clinical respiratory samples, herein we also describe a method by which wastewater surveillance can detect and determine mutations with a high likelihood of appearing in future VOCs. Society's ability to track and predict future mutations is important as the sequence of the infecting SARS-CoV-2 virus has been shown to affect the number of susceptible individuals and the efficacy of vaccines and therapeutics. For example, the evolution of the Omicron variant in November 2021 led to the largest surge in the COVID-19 pandemic to date, largely due to decreased immune efficacy and its appearance in winter months. Furthermore, subvariants of Omicron have been found to evade monoclonal antibody therapies, thereby denying infected individuals access to efficacious therapies. As SARS-CoV-2 will never cease to evolve and can do so in any area of the world, worldwide wastewater surveillance in the PEPP will allow us to monitor its evolution and prepare appropriately. Together, wastewater surveillance has been found to be a robust and effective tool necessary for success in the COVID-19 PEPP, and may also be used as an effective tool for other infectious diseases worldwide.

## Author contribution statement

Conceived and designed experiments - Bryan Sanchez Jimenez, Trinity Sterling, Austin Brown, Kristine N. Dye. Performed the experiments - Bryan Sanchez Jimenez, Trinity Sterling, Austin Brown, Brian Modica, Kaylee Gibson, Hannah Collins, Carolyn Koch, Tyler Schwarz, Kristine N. Dye. Analyzed and Interpreted the Data - Bryan Sanchez Jimenez, Trinity Sterling, Kristine N. Dye. Contributed reagents, materials, analysis tools or data - Kristine N. Dye. Wrote the paper - Bryan Sanchez Jimenez, Kristine N. Dye.

## Data availability

Data will be made available on request.

## Declaration of competing interest

The authors declare that they have no known competing financial interests or personal relationships that could have appeared to influence the work reported in this paper.
