# Supplementary material for: Wastewater surveillance in the COVID-19 post-emergency pandemic period: A promising approach to monitor and predict SARS-CoV-2 surges and evolution
Source: Heliyon. 2023 Nov 17;9(11):e22356. doi: 10.1016/j.heliyon.2023.e22356 (PMC10689941; doi:10.1016/j.heliyon.2023.e22356)
Supplement: Multimedia component 1 [file mmc1.pdf]

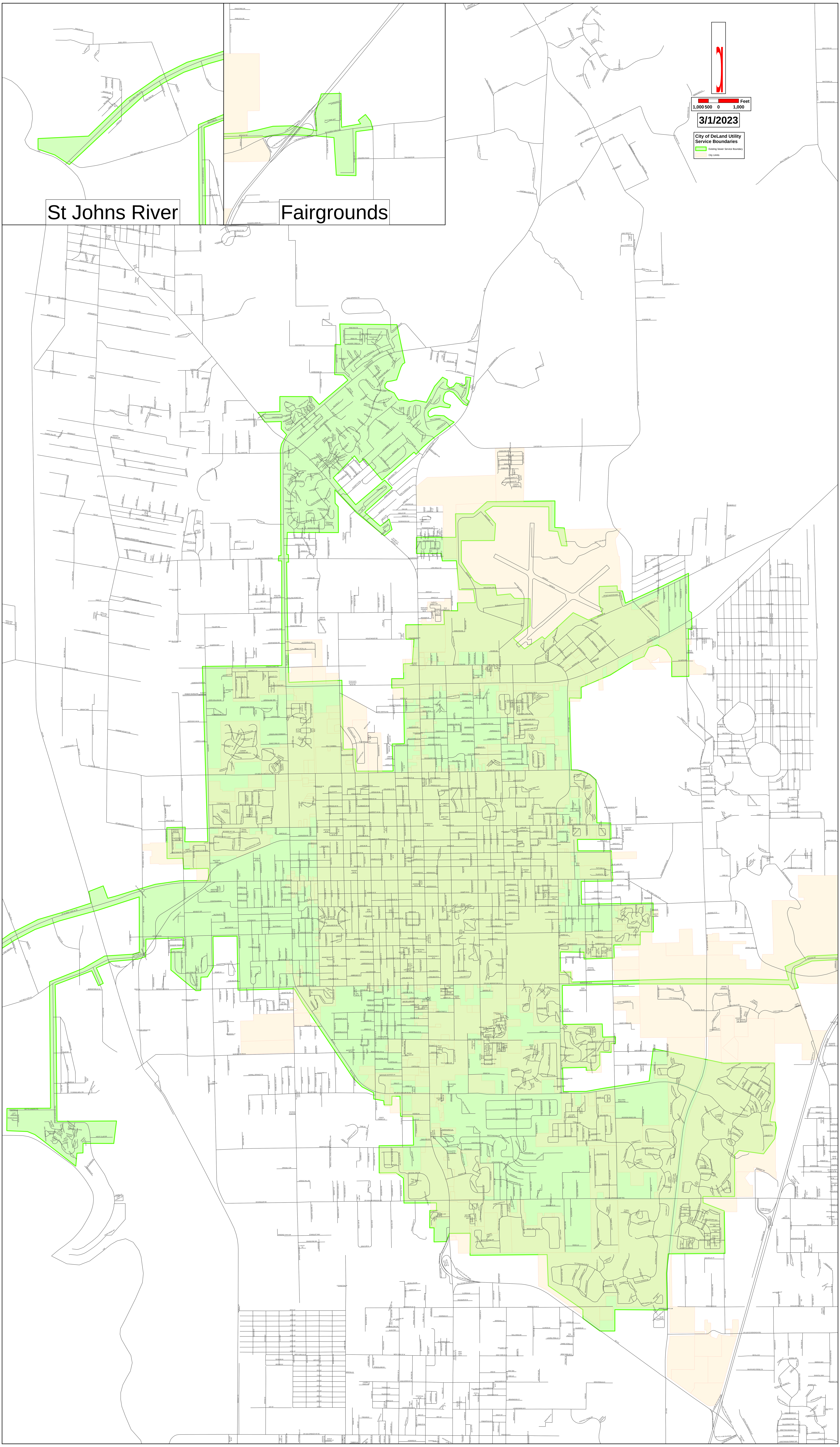
St Johns River
Fairgrounds
Feet
1,000 500 0 1,000
3/1/2023
City of DeLand Utility Service Boundaries
Existing Sewer Service Boundary
City Limits
